# FAP Expression in Renal Tumors Assessed by [^68^Ga]Ga-FAPI-46 PET Imaging and FAP Immunohistochemistry: A Case Series of Six Patients from the Prospective Exploratory Trial NCT04147494

**DOI:** 10.2967/jnumed.125.270346

**Published:** 2026-01

**Authors:** Adrien Holzgreve, Lena M. Unterrainer, Kimberly Flores, Ethan C. Lam, Christine E. Mona, Johannes Czernin, Brian M. Shuch, Anthony E. Sisk, Jeremie Calais

**Affiliations:** 1 Ahmanson Translational Theranostics Division, Department of Molecular and Medical Pharmacology, UCLA, Los Angeles, California;; 2 Department of Nuclear Medicine, LMU University Hospital, LMU Munich, Munich, Germany;; 3 Bavarian Cancer Research Center, partner site Munich, Munich, Germany;; 4 Department of Pathology, UCLA, Los Angeles, California;; 5 Jonsson Comprehensive Cancer Center, UCLA, Los Angeles, California; and; 6 Department of Urology, UCLA, Los Angeles, California

**Keywords:** kidney, renal cell carcinoma, RCC, oncocytoma, fibroblast activation protein inhibitor, NCT04147494

## Abstract

Fibroblast activation protein (FAP) has been proposed as a pan-tumor target for PET imaging using FAP-targeted tracers. Here, we explore the potential value of FAP PET in renal tumors. **Methods:** Six patients with renal tumors (4 with clear cell renal cell carcinoma, 1 with papillary renal cell carcinoma, and 1 with renal oncocytoma) who were included in a prospective imaging study (NCT04147494) underwent [^68^Ga]Ga-FAPI-46 PET before nephrectomy. FAP PET radiotracer uptake and FAP expression by immunohistochemistry were assessed in the tumors and surrounding renal parenchyma. **Results:** Tumoral FAP radiotracer uptake was highest in clear cell renal cell carcinoma (median SUV_max_, 3.1; range, 2.5–5.3), followed by renal oncocytoma (SUV_max_, 1.9) and papillary renal cell carcinoma (SUV_max_, 1.1). The FAP PET signal strongly correlated with FAP expression by immunohistochemistry (SUV_max_; *r* = 0.93; *P* = 0.007). **Conclusion:** FAP expression in different renal tumors, including renal cell carcinoma, was lower when compared with cancers with known FAP expression, such as sarcoma. Although our data do not favor FAP-based theranostic approaches in renal cell carcinoma, studies in larger cohorts are warranted for conclusive evidence.

## Visual Abstract

**Figure:**
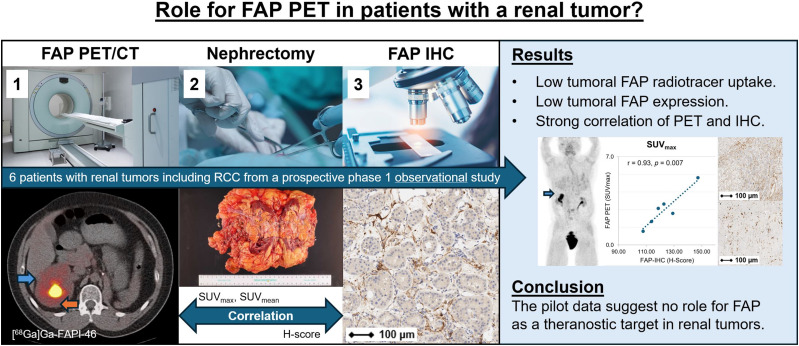

Fibroblast activation protein (FAP) is overexpressed on the surface of cancer-associated fibroblasts of the tumor stroma and can be visualized by PET using radiolabeled FAP tracers. FAP radiotracers demonstrate rapid accumulation in lesions, while the background signal on PET remains vastly low, a biodistribution feature that could be promising for the imaging of a variety of tumor entities (1,2). Beyond imaging, FAP has the potential to serve as a target for theranostic approaches (3) in many solid cancers. As such, it has been suggested that FAP can be leveraged as a target for PET imaging of and radiopharmaceutical therapy for renal cancer.

In this case series, we assessed the potential value of FAP PET for imaging malignant and benign renal tumors by analyzing uptake on [^68^Ga]Ga-FAPI-46 PET and correlating it with FAP expression measured by immunohistochemistry in excised tissue.

## MATERIALS AND METHODS

### Study Design and Participants

Six patients with renal tumors included in the prospective, exploratory, single-center imaging trial NCT04147494 who underwent [^68^Ga]Ga-FAPI-46 PET at a median of 10.5 d (range, 6–23 d) before a planned radical or partial nephrectomy between November 5, 2020, and June 13, 2023, were included in this analysis. [^68^Ga]Ga-FAPI-46 PET/CT imaging findings did not influence the therapy plan, and surgery was performed independently of the results of the scan findings. This study was approved by the University of California Los Angeles Institutional Review Board (19-000756). All patients provided oral and written informed consent.

[^68^Ga]Ga-FAPI-46 PET uptake and FAP expression by immunohistochemistry in surgically excised tissues were assessed and correlated in tumors and in the surrounding renal parenchyma on a per-patient basis.

### FAP PET/CT Imaging Acquisition

[^68^Ga]Ga-FAPI-46 was used as the radioligand (4). Details on PET/CT acquisition (5) are provided in the supplemental materials, available at http://jnm.snmjournals.org (6–8).

### FAP PET/CT Image Analysis

Images were analyzed in consensus by 2 nuclear medicine specialists who were masked to the immunohistochemistry results. The readers had access to all medical records and other available imaging results to facilitate tumor localization. Image analysis was performed with Visage 7 (Visage Imaging Inc.). The readers quantified [^68^Ga]Ga-FAPI-46 uptake on PET by placing a volume of interest in the solid parts of lesions as well as in the unaffected surrounding renal background (3-dimensional sphere with a minimum diameter of 1.5 cm). Volumes of interest were individually adapted to best encompass the structure of interest and to preclude spillovers from urine activity or other organs using anatomic information from CT or MRI. SUV_mean_ and SUV_max_ of the renal tumors, SUV_mean_ of the respective renal background, and lesion size (by CT) were recorded; the respective mean values of both readers were used for correlation with immunohistochemistry results.

### FAP Immunohistochemistry

Tissue sections (5 µm) were immunohistochemically stained for FAP, as previously described (9). Details regarding staining methods are provided in the supplemental materials. All preselected regions were used to run an automated quantification of FAP staining intensity and area distribution using Definiens software (MedImmune). The automated analysis resulted in an H-score per region, where H = (1 × %marker area low) + (2 × %marker area medium) + (3 × %marker area high). Per-patient mean scores were calculated across all regions within a lesion and across background tissue, respectively. These values were compared with PET parameters. H-scores used for immunohistochemical quantification of target expression ranged from 100 (lowest target expression) to 300 (highest target expression) (10).

### Statistical Analysis

Descriptive data were reported as median and range. Pearson correlation coefficient was used to assess linear correlations between FAP PET parameters and FAP immunohistochemistry scores. Statistical analysis was performed using Microsoft Excel for Mac version 16.94 (Microsoft) and SPSS Statistics version 29.0.0.0 (IBM). Because the subgroup sizes were small, no statistical tests for group comparisons (e.g., between distinct tumor types) were performed. A *P* value of less than 0.05 (two-sided) was considered statistically significant.

## RESULTS

Of the 6 patients included in the analysis, 4 had clear cell renal cell carcinoma (ccRCC), 1 had papillary RCC (pRCC), and 1 had renal oncocytoma. The median patient age was 57 y (range, 25–83 y). The median maximum lesion diameter for the entire cohort was 6.8 cm (range, 1.9–12.3 cm) and was highest in patients with ccRCC (8.1 cm), followed by oncocytoma (2.8 cm) and pRCC (1.9 cm). Patient characteristics and pathologic tumor characteristics are provided in Table 1 (11).

**TABLE 1.** Characteristics of Patients

Patient no.	Age (y)	Sex	Relevant comorbidity	Adjuvant or other prior treatment	Primary tumor location	Nephrectomy	Maximum size (cm)	Tumor type	WHO/ISUP grade	Pathologic stage
1	78	M	None	None	R upper	Radical	7.0	ccRCC	3–4	pT3a N0
2	45	M	None	4 cycles of ipilimumab + nivolumab	R middle	Radical	12.3	ccRCC	2–3	pT3a pM1
3	49	M	RPGN 3	None	R middle	Radical	9.2	ccRCC	2	pT3a N0
4*	83	M	None	Embolization for bleeding	L upper	Radical	6.6	ccRCC	3	pT3a N0
5	64	F	None	None	R upper	Partial	2.8	Oncocytoma	NA	NA
6	25	F	History of contralateral Wilms tumor	Contralateral nephrectomy + adrenalectomy 23 y ago	R upper	Partial	1.9	pRCC	2	pT1a NX

* Patient case 4 was previously published as a case report with a different scope (11).

WHO = World Health Organization; ISUP = International Society of Urological Pathology; RPGN 3 = rapidly progressive glomerulonephritis type 3 (i.e., pauci-immune disease); NA = not applicable.

**FIGURE 1.**
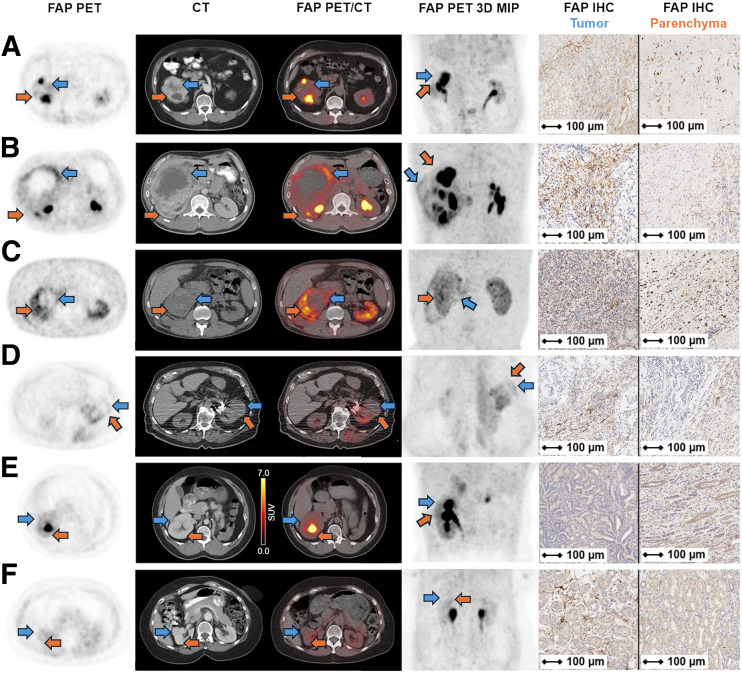
FAP PET/CT and FAP immunohistochemistry (IHC) results for patients with ccRCC (A–D), pRCC (E), and renal oncocytoma (F). Blue arrows point to tumoral lesions. Orange arrows point to renal parenchyma. MIP = maximum-intensity projection.

A pictorial comparison of PET and immunohistochemistry results is shown in Figure 1.

An atlas of all cases, including PET images, immunohistochemistry images, histopathologic features for each tumor and renal background, and a macroscopic gross photography of the surgically removed tumor, if available, is provided in the supplemental materials.

The median tumoral SUV_max_ on FAP PET of the entire cohort was 2.7 (range, 1.1–5.3). The median tumoral FAP radiotracer uptake was highest in patients with ccRCC (SUV_max_, 3.1), followed by oncocytoma (SUV_max_, 1.9) and pRCC (SUV_max_, 1.1). Strong uptake heterogeneity, perceived in the maximum-intensity-projection images, was related to urine activity versus kidney and tumor tissues and not to heterogeneity within the tumor tissues. The median H-score for tumoral FAP expression was 120.7 (range, 107.7–147.4). The median tumoral H-score was highest for ccRCC (126.0), followed by oncocytoma (113.9) and pRCC (107.7). One patient with rapidly progressive glomerulonephritis had a background SUV_mean_ of 3.5. The remaining 5 patients had a median background SUV_mean_ of 1.2 (range, 0.9–1.8). Individual PET and immunohistochemistry results are shown in Table 2 (11).

**TABLE 2.** Individual PET and Immunohistochemistry Results

	Tumor				Background	
Patient no.	Type	SUV_max_	SUV_mean_	H-score	SUV_mean_	H-score
1	ccRCC	3.2	2.0	122.7	1.2	134.0
2	ccRCC	5.3	3.3	147.4	1.8	135.8
3	ccRCC	2.9	1.2	118.8	3.5	127.6
4*	ccRCC	2.5	1.4	129.2	1.2	123.9
5	Renal oncocytoma	1.9	1.3	113.9	1.3	111.5
6	pRCC	1.1	1.0	107.7	0.9	101.4

* Patient case 4 was previously published as a case report with a different scope (11).

**FIGURE 2.**
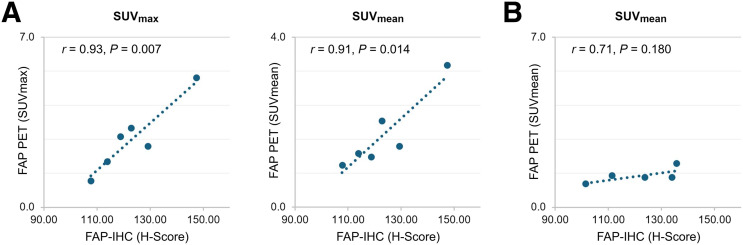
Correlation of FAP radiotracer uptake on PET with FAP expression by immunohistochemistry (IHC) for renal tumors (A) and renal parenchyma (B). Patient case with rapidly progressive glomerulonephritis and increased FAP radiotracer uptake in both kidneys was excluded in this figure.

Tumoral FAP radiotracer uptake on PET strongly correlated with the level of tumoral FAP expression, as determined by immunohistochemistry (*r* = 0.93 and *P* = 0.007 for tumoral SUV_max_ vs. tumoral H-score; *r* = 0.90 and *P* = 0.014 for tumoral SUV_mean_ vs. tumoral H-score). No such correlation was found for renal background tissue when including all cases (*r* = 0.40; *P* = 0.438). When excluding the single case of glomerulonephritis with markedly increased FAP radiotracer uptake in both kidneys, the FAP radiotracer uptake in the renal background for the remaining 5 patients tended to correlate with FAP expression in the renal background (*r* = 0.71; *P* = 0.180). The correlation of FAP radiotracer uptake on PET with FAP expression by immunohistochemistry in tumors and in renal background is shown in Figure 2.

## DISCUSSION

In this case series of 6 patients with renal tumors, preoperative FAP radiotracer PET uptake was low, consistent with low FAP expression in surgically excised tissues.

FAP PET has been suggested as a promising new diagnostic tool to address the limitations of previously available imaging modalities in renal cell carcinoma (RCC) (12,13). Before the advent of FAP PET imaging, detection of FAP in the tumor stroma of RCC by immunohistochemistry had been suggested for use as a biomarker for the presence of synchronous metastases and of metastases to the lymph nodes (14). In addition, FAP expression in the primary tumor correlated with a worse 10-y overall survival rate in patients with RCC (14), suggesting a potential role of FAP PET imaging for detecting more aggressive tumors with a higher likelihood for metastatic spread and shorter survival. Yet, our data suggest that FAP expression and FAP radiotracer uptake in renal tumors are mainly low, both by absolute values and in comparison with renal background uptake. Some uptake differences were noted between distinct tumors, with ccRCC cases showing the highest uptake and benign oncocytoma showing lower uptake, suggesting that FAP expression may be heterogeneous across renal tumors. An in-depth analysis stratified by tumor histology could not be performed because of the limited number of patients in this pilot cohort. When comparing absolute SUV, our findings are consistent with previously reported cases of FAP PET imaging using [^68^Ga]Ga-FAPI-04 in a larger RCC cohort (SUV_max_, 3.1 vs. 3.2), and a comparison with 28 types of cancer revealed RCC to have the lowest uptake, comparable to our results (2,12). Hence, our study supports previously published data and adds a direct correlation of in vivo imaging results with immunohistochemistry results in surgically resected tumors, confirming a low target expression. The renal background uptake on PET in 1 patient with glomerulonephritis was higher compared with the unaffected parenchyma of other patients. Although previous studies have reported higher FAP expression on FAP PET in diseases associated with renal fibrosis—such as [^68^Ga]Ga-FAPI-04 in lupus nephritis (SUV_mean_ of 2.8 vs. 1.5 in healthy controls (15)) and [^18^F]AlF-NOTA-FAPI-04 in IgA nephropathy (SUV_mean_ of 3.6 vs. 1.5 in controls (16))—this was not reflected in the immunohistochemistry results of our patient with rapidly progressive glomerulonephritis type 3. This suggests additional contributors to the higher PET signal in this case, such as unspecific mechanisms related to kidney failure (17). Another contributing factor may be the limited representativeness of our immunohistochemistry results for healthy kidney parenchyma, as fewer slides were available for analysis compared with tumor tissue.

Compared with other emerging targets for diagnostic and theranostic approaches including carbonic anhydrase 9–directed PET with a reported SUV_max_ of more than 150 in RCC cases (18), our data suggest FAP to be of subordinate value in renal tumors (19).

## CONCLUSION

FAP expression in different renal tumors, including RCC, was lower when compared with cancers with known FAP expression, such as sarcoma. Although our data do not favor FAP-based theranostic approaches in RCC, studies in larger cohorts are warranted for conclusive evidence.

## Notes

KEY POINTS **QUESTION:** Is there a role for FAP PET in patients with a renal tumor? **PERTINENT FINDINGS:** Renal tumors showed low FAP radiotracer uptake on PET and low FAP expression in excised tissue. PET and immunohistochemistry results were strongly correlated. **IMPLICATIONS FOR PATIENT CARE:** The pilot data do not favor a role for FAP as a theranostic target in renal tumors.
